# Apelin Ameliorates High Glucose-Induced Downregulation of Connexin 43 via AMPK-Dependent Pathway in Neonatal Rat Cardiomyocytes

**DOI:** 10.14336/AD.2017.0426

**Published:** 2018-02-01

**Authors:** Xiaoting Li, Lu Yu, Jing Gao, Xukun Bi, Juhong Zhang, Shiming Xu, Meihui Wang, Mengmeng Chen, Fuyu Qiu, Guosheng Fu

**Affiliations:** ^1^Department of Cardiology, Sir Run Run Shaw Hospital, Zhejiang University School of Medicine, Hangzhou, Zhejiang, China; ^2^Zhejiang University School of Medicine, Hangzhou, Zhejiang, China; ^3^Institute of Translational Medicine, Zhejiang University School of Medicine, Hangzhou, Zhejiang, China; ^4^Biomedical Research Center, Sir Run Run Shaw Hospital, Zhejiang University School of Medicine, Hangzhou, Zhejiang, China

**Keywords:** cardiomyocytes, connexin 43, high glucose, apelin, gap junction, AMPK

## Abstract

Diabetes Mellitus is a common disorder, with increasing risk of cardiac arrhythmias. Studies have shown that altered connexin expression and gap junction remodeling under hyperglycemia contribute to the high prevalence of cardiac arrhythmias and even sudden death. Connexin 43 (Cx43), a major protein that assembles to form cardiac gap junctions, has been found to be downregulated under high glucose conditions, along with inhibition of gap junctional intercellular communication (GJIC). While, apelin, a beneficial adipokine, increases Cx43 protein expression in mouse and human embryonic stem cells during cardiac differentiation. However, it remains unknown whether apelin influences GJIC capacity in cardiomyocytes. Here, using Western blotting and dye transfer assays, we found that Cx43 protein expression was reduced and GJIC was impaired after treatment with high glucose, which, however, could be abrogated after apelin treatment for 48 h. We also found that apelin increased Cx43 expression under normal glucose. Real-time PCR showed that the Cx43 mRNA was not significantly affected under high glucose conditions in the presence of apelin or high glucose and apelin. High glucose decreased the phosphorylation of AMPKα; however, apelin activated AMPKα. Interestingly, we found that Cx43 expression was increased after treatment with AICAR, an activator of AMPK signaling. AMPKα inhibition mediated with transfection of siRNA-AMPKα1 and siRNA-AMPKα2 abolished the protective effect of apelin on Cx43 expression. Our data suggest that apelin attenuates high glucose-induced Cx43 downregulation and improves the loss of functional gap junctions partly through the AMPK pathway.

Diabetic cardiac dysfunction is characterized by mechanical and electrical abnormalities, which, in turn, result in a high prevalence of cardiac arrhythmias and sudden death [1]. Altered connexin expression and gap junction remodeling contribute to the high susceptibility of diabetic hearts to arrhythmias [2, 3]. Connexin 43 (Cx43) is the most extensive expressed connexin that assembles to form gap junctions in mammalian ventricular muscle [4-6]. Abnormal Cx43 expression and location were found in hypertrophic, ischemic and diabetic hearts [7-10]. Accordingly, hyperglycemia is a pivotal initiator for diabetic cardiac complications [11], and previous studies have reported that gap junctional intercellular communications (GJICs) are impaired and Cx43 expression is reduced in neonatal rat cardiomyocytes (NRCMs) exposed to high glucose [12, 13].

Apelin is a beneficial adipokine that is widely present in numerous tissues, such as the heart, lung, kidney, liver, and adipose tissue. Tatemoto, et al. firstly found the endogenous ligand named apelin for Angiotensin II receptor-like 1 (APJ) from bovine stomach homogenates in 1998 [14]. Apelin gene encodes for a 77-amino acid pre-propeptide, and prepro-apelin can be cleaved into different bioactive apelin fragments, including apelin-36, apelin-17 and apelin-13 [15]. Among them, apelin-13 is predominant in the heart [16, 17]. Apelin/APJ system has been considered to play a key role in various physiological processes, such as energy metabolism, angiogenesis, cardiovascular functions and fluid homeostasis [17, 18]. Experimental evidence from various studies has indicated that apelin potently improves cardiac contractility [19-21] and alleviates ischemia-reperfusion injury [22, 23]. Generally, the cardioprotective effect of apelin in diabetic hearts is that apelin stimulates neovascularization, improves glucose uptake and insulin sensitivity in cardiomyocytes [24]. Several studies observed low plasma levels of apelin in patients with atrial fibrillation [25-27]. During cardiac differentiation of mouse and human embryonic stem cells, the increased expression of Cx43 was found in group treated with apelin [28]. However, it remains unclear whether apelin can treat arrhythmias.

Adenosine monophosphate-activated protein kinase (AMPK) is a serine-threonine kinase composed with three different subunits: a catalytic subunit (α), a scaffolding subunit (β) and a regulatory subunit (γ). AMPK regulates energy metabolism and many other cellular processes involved in overall cell health, such as cell growth, autophagy, apoptosis, and regulation of cardiac sodium and potassium channels [29-31]. AMPK also plays an important adaptive role in a variety of arrhythmia-promoting cardiovascular diseases and can modify arrhythmogenic conditions [32]. Moreover, several studies have demonstrated that Cx43 expression was regulated by AMPK activity [29, 33, 34].

Even though apelin has been found to increases Cx43 expression in embryonic stem cells, whether apelin improves GJIC and the underlying mechanisms remain unknown. In this study, we aimed to identify the effects of apelin on the expression and function of gap junctions in NRCMs under high glucose treatment and to investigate whether AMPK was implicated in this condition.

## MATERIALS AND METHODS

### Chemicals and Reagents

Apelin-13, glucose, mannitol, Lucifer Yellow CH dilithium salt, and Rhodamine-dextran were purchased from Sigma (St. Louis, USA). AICAR was purchased from Selleck (Houston, USA). Dulbecco’s modified Eagle’s medium(DMEM) and Opti-MEM (reduced serum media) were obtained from GIBCO (Grand Island, NY, USA). Newborn calf serum (NBCS) was supplied by Tianhang Biotechnology (Hangzhou, China). Polyclonal rabbit antibodies including anti-Cx43 antibody, anti-AMPKα, and anti-phospho-AMPKα, were obtained from Cell Signaling Technology (Massachusetts, USA). β-tubulin monoclonal antibody (HRP conjugated) was purchased from MultiSciences (Hangzhou, China).

### Cell Culture and Treatment

NRCMs were prepared from 1-to-2-day-old Sprague-Dawley rats (provided by the Experimental Animal Center of Zhejiang Province), and the animal experiments were approved by the Institutional Animal Care and Use Committee of Zhejiang University. NRCMs were isolated and cultured as previously described [12]. Breifly, hearts were dissected and digested in 0.12% trypsin and 0.1% collagenase II at 37°C. The cell suspension was then cultured in DMEM (5.5 mM glucose) with 10% (v/v) NBCS in a humidified 37°C, 5% CO_2_ incubator (Thermo, MA, USA). The fibroblasts were deducted from the cell suspension by pre-plating for 90 min on account of differential cell adhesion. Cells were seeded 1×10^6^/well in 6-well plates. Cell culture medium was replaced every 48 h. Apelin-13 was dissolved in sterilized phosphate buffer solution (PBS, pH 7.4) and used for treating cells in various concentrations as indicated. Mannitol (24.5 mM) was dissolved in DMEM (5.5 mM glucose) as an osmotic control.

### Western Blotting Analysis

Cells in 6-well plates were extracted in a radioimmunoprecipitation assay (RIPA) lysis buffer containing 1 mM phenylmethylsulfonyl fluoride (PMSF). Proteins were quantified with a bicinchoninic acid (BCA) assay kit (Beyotime Biotechnology, Shanghai, China). Samples containing equal amounts of protein were separated using 10% SDS polyacrylamide gels, transferred onto polyvinylidene difluoride membranes (PVDF, Bio-Rad, CA, USA), and blocked for 1 h in Tris-buffered saline and 0.1% Tween 20 (TBST) with 5% (w/v) nonfat dry milk at room temperature. Next, PVDF membranes were incubated with primary antibodies overnight at 4°C followed by HRP-linked secondary antibodies for 1 h at room temperature after 3 TBST washes. The bands were exposed by reacting with enhanced chemiluminescence (ECL) reagents on an Amersham Imager 600 system (GE Healthcare, Buckinghamshire, England).

**Figure 1.**
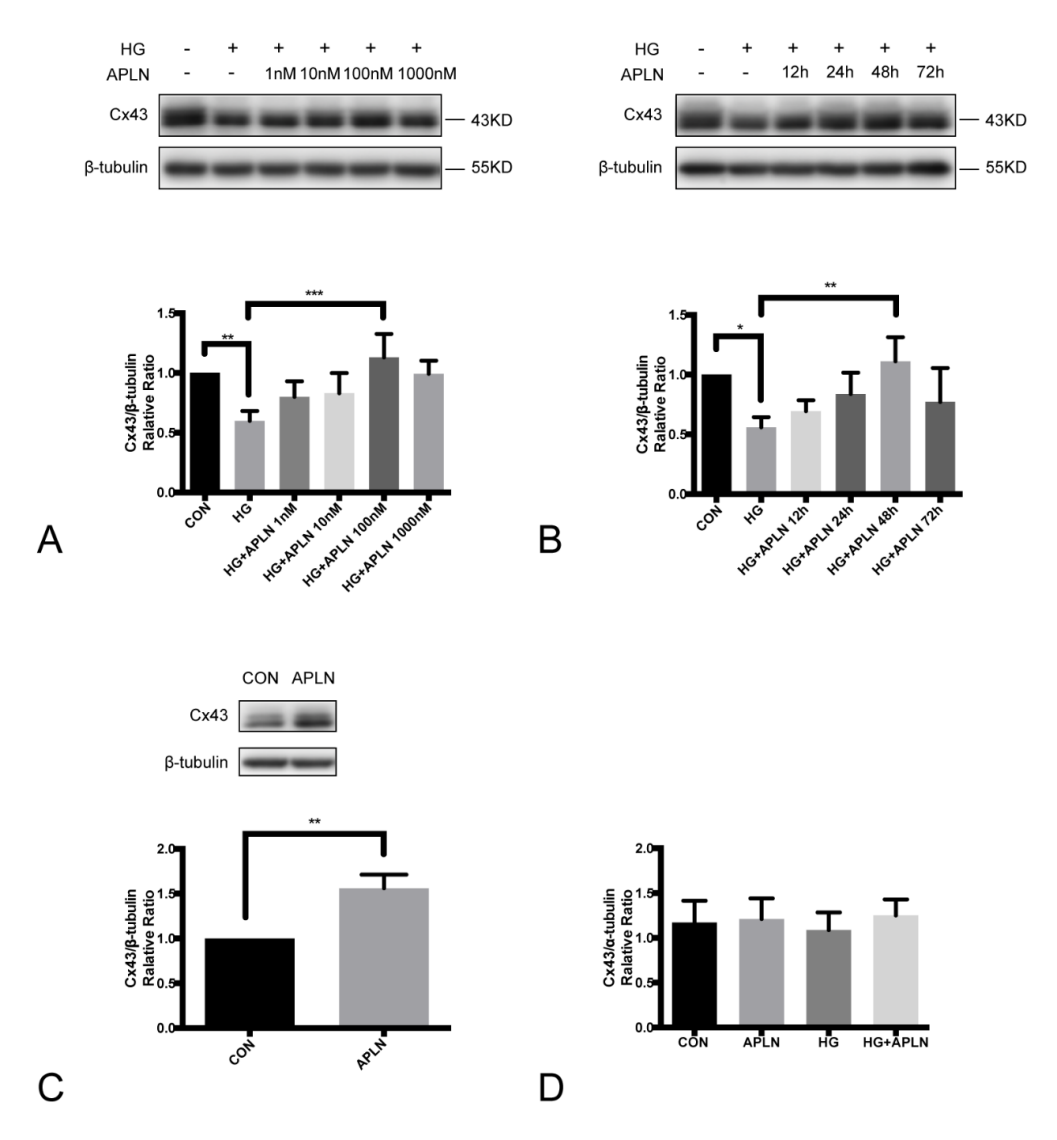
Effects of apelin-13 on Cx43 expression in NRCMs Apelin-13 reversed the Cx43 downregulation triggered by high glucose in a concentration- (**A**) and time-dependent (**B**) manner. The expression of Cx43 significantly decreased under high glucose condition (30 mM, 72 h). The addition of apelin-13 (100 nM, 48 h) apparently alleviated the Cx43 reduction induced by high glucose. (**C**) Incubation with 100 nM apelin-13 only for 48 h increased the Cx43 protein expression. (**D**) The effects of high glucose and/or apelin-13 on Cx43 mRNA level were quantified by real-time PCR. CON: normal glucose (5.5 mM); HG: high glucose (30 mM); APLN: apelin-13 (100 nM). *P<0.05, **P<0.01, ***P<0.001. Data are the mean ± SD.

**Figure 2.**
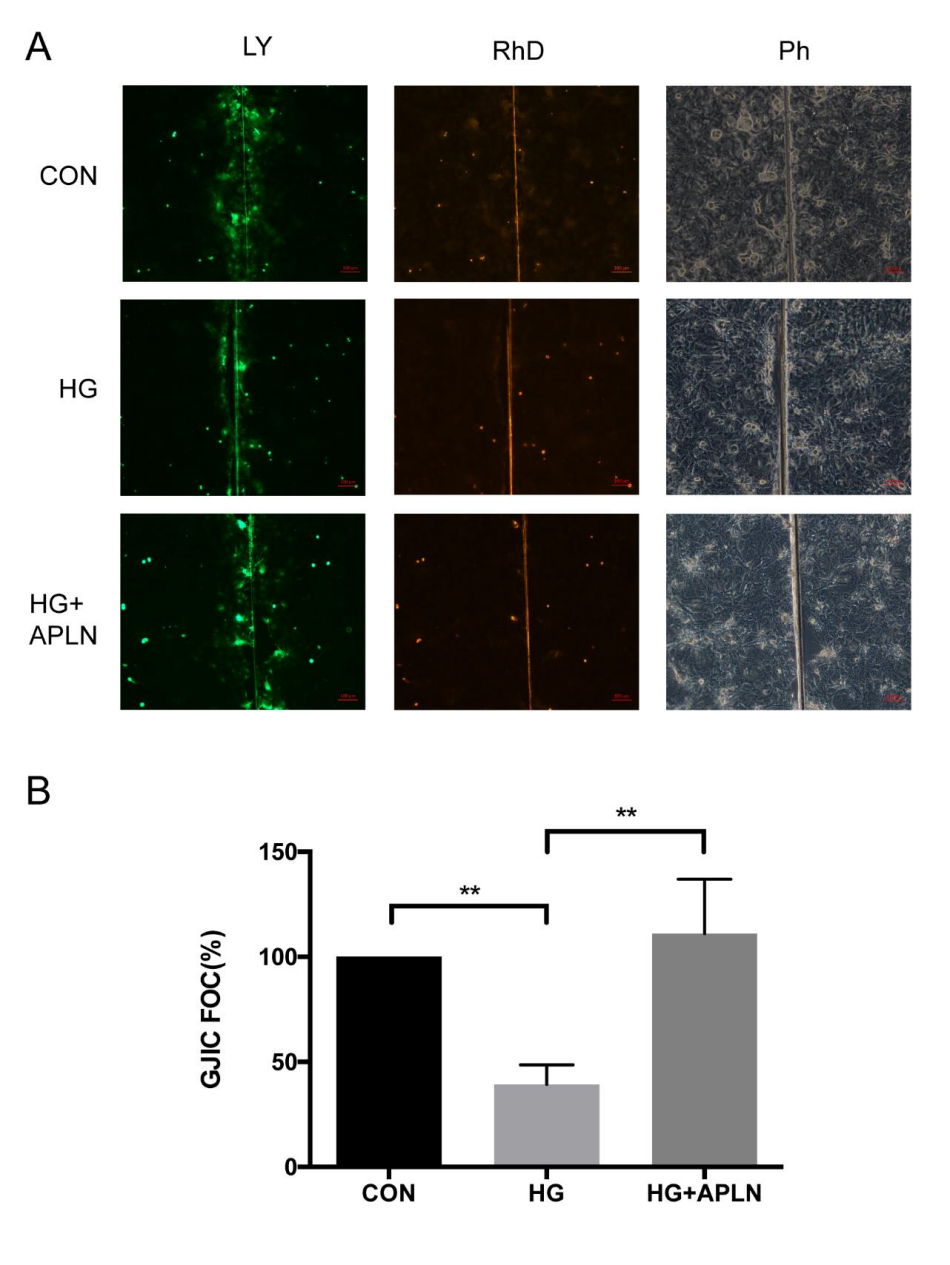
Effects of apelin-13 on high-glucose induced GJIC in NRCMs The GJIC capacity of NRCMs was evaluated by dye transfer assay. (**A**) Cells were left untreated in the control group. (**B**) Cells were incubated with 30 mM glucose for 72 h. (**C**) Cells were initially treated with 30 mM glucose for 24 h and then co-treated with 100 nM apelin-13 for the following 48 h. (**D**) The statistical data of net LY dye transfer area indicated that high glucose significantly reduced the LY transfer area and that apelin-13 rescued the high glucose-induce transfer area to a normal level. CON: normal glucose (5.5 mM); HG: high glucose (30 mM); APLN: apelin-13 (100 nM). *P<0.05, **P<0.01, ***P<0.001. Data are the mean ± SD.

### qRT-PCR

Total RNA was extracted with TRIZOL reagent (CWBIO, Beijing, China) and converted into cDNA using a PrimeScript RT reagent Kit (Takara, Tokyo, Japan) according to the manufacturer’s instructions. Real-time PCR was performed on the Viia 7 system (Applied Biosystems, CA, USA) using UltraSYBR Mixture (Low ROX) (CWBIO, Beijing, China). The following primers were used: Cx43 forward, 5′-TCTGCCTTTC GCTGTAACACT-3′, reverse, 5′-GGGCACAGACAC GAATATGAT-3′; α-tubulin forward, 5′-GACGGA TGCAGAAGGAGATTACT-3′, reverse, 5′-TGATCCA CATCTGCTGG AAGGT-3′.

### Scrape Loading/Dye Transfer Assay

The scrape loading/dye transfer (SL/DL) assay was used to assess gap junctional intercellular communication (GJIC). Lucifer yellow (LY, MW 457), a membrane impermeable fluorescent dye that can go across junctional channels, is the most popular dye in research use. Rhodamine-dextran (Rhd, MW 10000), which is too large to transverse the junctional channels, is often used as an additional control to identify the cells initially loaded after the scrape [35]. After treatment with high glucose and apelin, cells seeded in 35 mm dishes were rinsed gently three times with PBS (pre-warmed to 37°C). Sufficient 1 mg/mL LY-dye and 1 mg/mL rhodamine-dextran mixed solution (37°C) was added to the dishes, and then the cells were scraped with a surgical steel blade. After incubation at 37°C for 4 min, the cells were washed three times with warmed PBS and then fixed by adding 4% paraformaldehyde before visualization with a fluorescent microscope (Zeiss, Oberkochen, Germany). National Institute of Health (NIH) imaging system was used to calculate the fluorescence area. The fraction of the control (GJIC-FOC) was used to evaluate the GJIC capacity [35].

GJIC FOC_Treatment_ = (Area_Treatment_^LY^ - Area_Treatment_^Rhd^) / (Area_Control_^LY^ - Area_Control_^Rhd^)

### Transfection of siRNA

The siRNAs against AMPKα1 and AMPKα2 were designed and synthesized by Sigma (St. Louis, USA). An unsilencing siRNA was used as a negative control. The siRNA-AMPKα1 and siRNA-AMPKα2 sequence were as follows: siRNA-AMPKα1 sense strand, 5′-CUAUGAAU GGAAGGUUGUAdTdT-3′, anti-sense strand, 5′-UAC AACCUUCCAUUCAUAGdTdT-3′; siRNA-AMPKα2 sense strand, 5′-GCUUUACCUGGUUGACAAUdTdT-3′, anti-sense strand, 5′-AUUGUCAACCAGGUAAAG CdTdT-3′.

The siRNA and Hiperfect transfection reagents (Qiagen, Hilden, Germany) were premixed with Opti-MEM at room temperature for 10 min. Cells in 6-well plates were incubated with 500 μL of mixed solution containing 100 nM siRNA for 6 h and then added to 1500 μL of DMEM. High glucose and apelin were added 48 h after the transfection.

**Figure 3.**
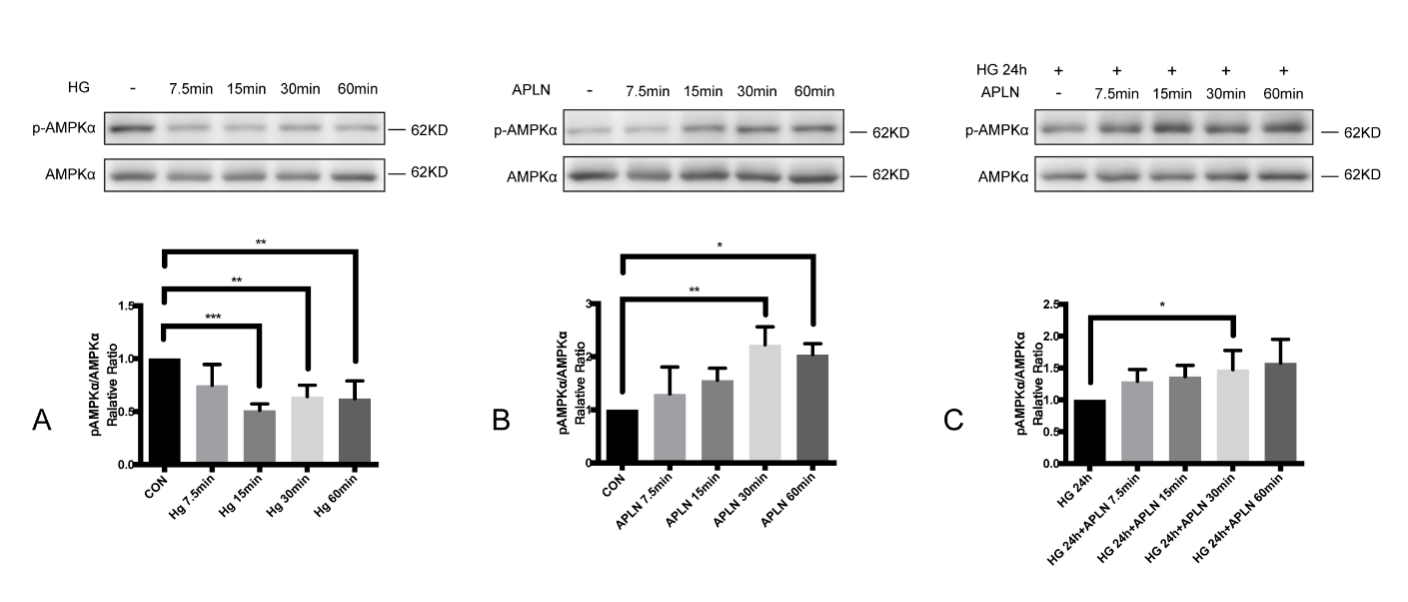
The activity of AMPK induced by high glucose and apelin in NRCMs (**A**) Cells were separately treated with 30 mM glucose for 7.5 min, 15 min, 30 min and 60 min. The p-AMPKα/AMPKα ratio declined significantly after high glucose treatment for 7.5 min. (**B**) The phosphorylation of AMPKα increased after the administration of apelin-13 for 30min. (**C**) Cells were pre-treated with 30 mM glucose for 24h, and the level of pAMPKα/AMPKα increased at 30 min after adding apelin. CON: normal glucose (5.5 mM); HG: high glucose (30 mM); APLN: apelin-13 (100 nM). *P<0.05, **P<0.01, ***P<0.001. Data are the mean ± SD.

### Statistical Analysis

Results were expressed as mean ± SD, and experiments were repeated for at least three times. The statistical analysis was performed using one-way ANOVA. Significant differences were considered at *P* < 0.05.

## RESULTS

### Apelin-13 attenuated the Cx43 downregulation induced by high glucose in NRCMs.

Cells were pre-incubated with 30 mM high glucose for 24 h, and then co-treated with apelin-13 at the indicated concentrations (1 nM, 10 nM, 100 nM, 1000 nM) for the following 48 h. Apelin-13 was demonstrated to reverse the Cx43 protein reduction in a dose-dependent manner with a peak at 100 nM (Fig. 1A). NRCMs were treated with apelin-13 (100 nM) at various times (12, 24, 48, and 72 h) under 30 mM high glucose conditions for 72 h. The expression of Cx43 was reduced under high glucose, and the decrease was mitigated by treatment with apelin-13 in a time-dependent manner with a peak at 48 h. The protective effect of apelin seemed to evaporate at 72 h (Fig. 1B). In cells treated with apelin-13 (100 nM, 48 h) only, Cx43 expression was significantly increased as well (Fig. 1C). However, the level of Cx43 mRNA was not affected by either high glucose or co-treatment with apelin-13 (Fig. 1D).

### Apelin-13 improved the high glucose-induced GJIC.

To examine the influence of apelin-13 on the function of gap junctions, a dye transfer assay was applied to measure the GJIC. In the control group, cells were found to remain well coupled by monitoring the net Lucifer Yellow areas. Under high glucose conditions (30 mM, 72 h), the net LY transfer area was significantly reduced. However, the addition of apelin-13 (100 nM, 48 h) to cells effectively ameliorated the reduced area caused by high glucose (Fig. 2A). The quantitative data of net LY transfer area indicated that half of the area reduced under high glucose condition was rescued to normal levels following apelin treatment (Fig. 2B). These results indicated that apelin-13 can protect the GJIC capacity from high glucose conditions.

**Figure 4.**
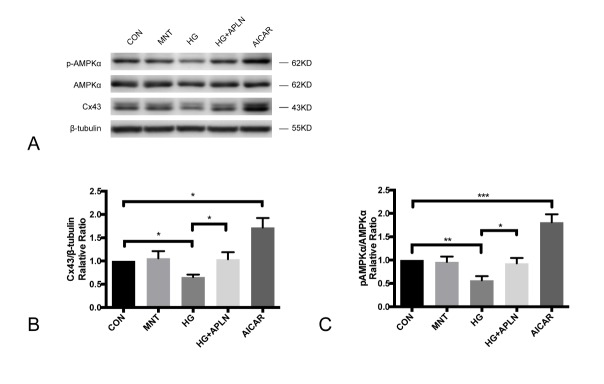
The effect of AICAR on Cx43 and pAMPK expression in NRCMs (**A**) Cells incubated under normal glucose (CON), osmotic control (MNT), high glucose (HG), high glucose with apelin (HG+APLN) and AICAR were harvested for western blotting. (**B**) and (**C**) Compared with control group, the Cx43 expression and pAMPK/AMPKα ratio in osmotic control group were not significantly different. The expression and of Cx43 and pAMPK in high glucose group (30 mM, 72h) was significantly reduced compared with control group, and co-treatment with apelin (100 nM, 48h) relieved the downregulated Cx43 and pAMPK expression. Compared with control group, pAMPKα and Cx43 expression was significantly increased in AICAR group.CON: normal glucose (5.5 mM); MNT: normal glucose (5.5 mM) and mannitol (24.5 mM); HG: high glucose (30 mM); APLN: apelin-13 (100 nM). AICAR: an AMPK activator (0.5 mM). *P<0.05, **P<0.01, ***P<0.001. Data are the mean ± SD.

**Figure 5.**
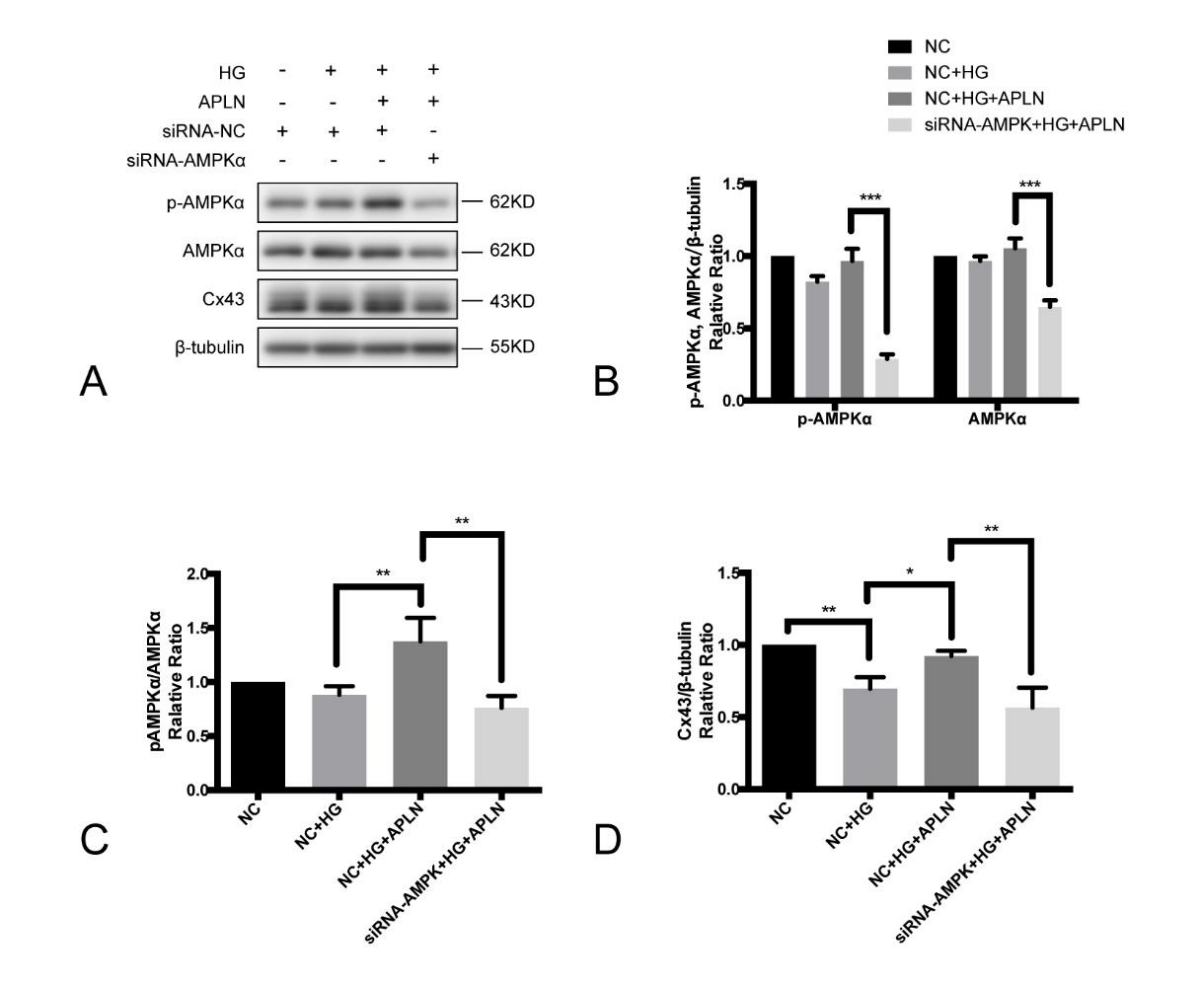
AMPKα suppression inhibited Cx43 expression up-regulated by apelin-13 (**A**) Western blotting was examined to reveal the expression of p-AMPKα, AMPKα and Cx43 in cells transfected with siRNA-NC and siRNA-AMPKα1/α2. During the transfection of siRNA, cells were cultured under normal glucose. After 48 h, cells were cultured in high glucose conditions for 72 h with/without apelin (100 nM, 48 h). (**B**) siRNA-AMPKα1/α2 significantly suppressed the expression of p-AMPKα and AMPKα compared with negative control siRNA. (**C**) Compared with the high glucose group, apelin-13 addition could increase the p-AMPKα/AMPKα ratio in cells transfected with siRNA-NC, while siRNA-AMPKα1/α2 reduced the p-AMPKα/AMPKα ratio. (**D**) In cells transfected with negative control siRNA, Cx43 expression was reduced under high glucose and retained with apelin-13 co-treatment. The effect of apelin-13 on Cx43 was abolished by siRNA-AMPKα1/α2. SiRNA-NC: negative control siRNA; *P<0.05, **P<0.01, ***P<0.001. Data are the mean ± SD.

### High glucose inhibited AMPK activity while apelin-13 activated AMPK in NRCMs.

To investigate the alterations of AMPK activity by stimulation, cells were treated with high glucose (30 mM) or apelin-13 (100 nM) for 7.5 min, 15 min, 30 min and 60 min. Western blotting analysis showed that the ratio of p-AMPKα to AMPKα protein significantly decreased under high glucose stimulation and increased under apelin-13 interference (Fig. 3A and 3B). AMPKα activation by apelin-13 remained in cells pre-treated with high glucose (30 mM) for 24 h (Fig. 3C).

### AICAR upregulated pAMPKα and Cx43 expression in NRCMs.

Since we found that apelin-13 could increase AMPKα, AICAR (an AMPK activator) was used as positive control. NRCMs were treated with AICAR (0.5mM) for 48h to observe the AMPK activity and Cx43 expression. Mannitol here was an osmotic control. The Cx43 expression and pAMPK/AMPKα ratio between control and mannitol group was similar. High glucose (30 mM, 72h) apparently reduced Cx43 and pAMPKα expression while the addition of apelin (100 nM, 48h) relieved the decline. Compared with the control group, AICAR significantly stimulated AMPKα phosphorylation and enhanced Cx43 expression (Fig. 5). These data further implied the correlation between AMPK activity and Cx43 expression.

### Silenced AMPKα expression inhibited Cx43 expression and abolished the protective effect of apelin-13.

To determine the role of AMPK in Cx43 expression, RNA interference technology was employed to suppress AMPKα expression. Western blotting was applied to show the expression of p-AMPKα, AMPKα and Cx43 in cells transfected with the negative control siRNA and siRNA-AMPKα1/α2 (Fig. 5A). In cells co-treated with high glucose and apelin-13, siRNA-AMPK notably repressed the expression of AMPK and phospho-AMPK compared with the negative control siRNA (Fig. 5B). siRNA-AMPKα1 and siRNA-AMPKα2 reduced the ratio of p-AMPKα to AMPKα protein and abolished the Cx43 abundance induced by apelin-13 (Fig. 5C and 5D). All these observations indicated that the AMPK signaling pathway might be partially involved in the protective effect of apelin-13 on Cx43 protein expression reduced by high glucose.

## DISCUSSION

A novel feature of our study is that we identified the protective effect of apelin-13 on gap junctions in cultured cardiomyocytes. Previous studies [12, 13] have reported that the expression of Cx43 was reduced by high glucose in neonatal rat cardiomyocytes. In this work, we found that although either high glucose or apelin-13 did not affect the level of Cx43 mRNA, apelin-13 attenuated the high glucose-induced Cx43 protein down-regulation and functional impairment of GJIC. The AMPK signaling pathway might be implicated in this progress. This conclusion was supported by the findings that high glucose inhibited AMPKα, while apelin-13 activated the phosphorylation of AMPKα, and silenced AMPKα expression abolished the protective effect of apelin-13.

The diminished expression and heterogeneous distribution of Cx43 impaired the function of gap junctions, resulting in conduction defects in the heart [36, 37]. In streptozotocin (STZ)-induced diabetic rat models, the expression of Cx43 fluctuated in several studies with differences in STZ concentrations and durations of STZ injections. High glucose contributed to the decrease of Cx43 in a 3-week STZ-induced diabetic rat model [38, 39]. However, the increase of Cx43 in a 12-week diabetic rat model [7] may have been resulted from accumulated advanced glycation end products (AGEs) from long-term complications of diabetes mellitus [40]. Additionally, our previous study detected Cx43 up-regulation and lateralization in AGE-infused rats [41]. Considering the complexity of animal models, NRCMs under high glucose (30 mM, mimic of hyperglycemia) were utilized to investigate the effect of apelin on Cx43 expression in the present study.

Apelin, a small peptide, plays important roles in energy metabolism, regulation of cardiac function and improvement of insulin sensitivity [18]. It has also been demonstrated to induce a positive inotropic response in mouse hearts [19-21]. Cx43 protein expression is increased in mouse and human embryonic stem cells treated with apelin [28]. Our data were consistent with the effect of apelin on Cx43 up-regulation, and the dye transfer assay indicated that apelin was capable of improving gap junctional communication in cardiomyocytes under high glucose. Since the Cx43 transcript level did not vary with the addition of either high glucose or apelin, we hypothesized that apelin amended Cx43 protein expression by post-transcriptional regulation, protein translation or protein degradation.

Apart from its key role in regulating energy metabolism, AMPK is also a fundamental regulator of cellular proteostasis [42]. AMPK is composed of a catalytic subunit (α1 and α2), a scaffolding subunit (β1 and β2) and a regulatory subunit (γ1, γ2) in cardiac tissue. The α1- and α2-subunits are encoded by PRKAA1 and PRKAA2, respectively, and threonine 174 (Thr174) on the α1-subunit and Thr172 on the α2-subunit regulate AMPK activation [30, 43]. In addition, AMPK has been identified as a major downstream signaling molecule of Apelin [22]. Previous studies demonstrated that AMPK activated mitochondrial K_ATP_ channels, thus increasing Cx43 expression, improving its distribution and attenuating the arrhythmogenic response to programmed electrical stimulation [44]. And Cx43 was found to be reduced in LKB1 (a protein kinase that activates AMPK) deletion mice.

In accordance with the activated AMPK signaling pathway in type II diabetic mice treated with apelin-13 [45, 46], we also found that apelin-13 stimulated AMPKα phosphorylation in NRCMs. In this case, we hypothesized that the protective effect of apelin on Cx43 expression might be mediated, at least in part, via the AMPK pathway. In our study, high glucose inhibited the phosphorylation of AMPKα in a time-dependent manner while apelin activated AMPKα in NRCMs. The impact of apelin on AMPKα still existed in myocardiocytes pre-incubated with high glucose for 24 h. The widely-used inhibitor of AMPK, dorsomorphin (also called Compound C), is a selective inhibitor of bone morphogenetic protein (BMP) signaling [47]. BMP4, BMP7 and BMP15 significantly decreased Cx43 expression in human granulosa cells [48, 49]. Considering that dorsomorphin could increase Cx43 expression by inhibiting BMP signaling, it was not an applicable AMPK inhibitor in this study. Our study detected that siRNA-AMPKα1 and siRNA-AMPKα2 could suppress AMPKα expression and abrogate the up-regulation of Cx43 expression by apelin.

In consistent with our findings, Guo, et al. observed that expression of Cx43 and p-AMPK was diminished in diabetic nephropathy specimens and cultured glomerular mesangial cells (GMCs) under high glucose conditions. In cultured GMCs, silenced AMPK expression by siRNA suppressed Cx43 expression while AMPK activation with metformin alleviated the down-regulation of Cx43 induced by high glucose [34]. However, recent data from Alesutan, et al. suggested that AMPKα1 stimulated ubiquitination of Cx43, thereby leading to decreased Cx43 protein abundance in transverse aortic constriction (TAC)-treated mouse cardiac tissue [29]. Possible reasons for the controversial outcomes might be from the different animal models. The fundamental level of AMPK activity varies according to the models of diseases. As previously mentioned, AMPK was stimulated in mice exposured to overload pressure, whereas AMPK activity was inhibited in high glucose-treated GMCs and H9C2 cardiac myoblast cells [50]. The action of AMPK, whether beneficial or detrimental, remains uncertain as well. We presumed that it was determined by the maintenance of efficient cellular homeostasis.

In summary, this study shed new light on the potential role of apelin in protecting the cardiac gap junctions from remodeling under hyperglycemia. Apelin attenuated high glucose-triggered Cx43 reduction and improved the function of gap junctions partly through the AMPK pathway. This suggests that apelin-13 might be a novel therapeutic strategy, via regulation of AMPK activity, for arrhythmias in diabetic cardiopathy. Further studies are needed to determine the downstream molecules of AMPK involved in Cx43 alteration and the impact of apelin on gap junctions in animal models.
